# Tumor Lysis Syndrome with Venetoclax/Carfilzomib/Dexamethasone for Relapsed/Refractory Multiple Myeloma: A Case Report

**DOI:** 10.3390/reports7040108

**Published:** 2024-11-29

**Authors:** Reilly Fankhauser, Alan Lu, Adetola Kassim, Eden Biltibo

**Affiliations:** 1 Medical Scientist Training Program, Vanderbilt University Medical Center, Nashville, TN 37232, USA; reilly.g.fankhauser@vanderbilt.edu; 2 Department of Pathology, Microbiology and Immunology, Vanderbilt University Medical Center, Nashville, TN 37232, USA; 3 Division of Hematology/Oncology, Department of Medicine, Vanderbilt University Medical Center, Nashville, TN 37232, USA

**Keywords:** relapsed refractory multiple myeloma (RRMM), tumor lysis syndrome (TLS), venetoclax, BCL-2, t(11;14), carfilzomib, dexamethasone

## Abstract

**Background and Clinical Significance:** Tumor lysis syndrome (TLS) is a rare occurrence in patients treated with venetoclax mono- or combination therapy, and clear protocols guiding TLS prophylaxis are lacking. **Case Presentation:** We present a 53-year-old male with a history of relapsed refractory multiple myeloma (RRMM) with t(11;14) treated with venetoclax, carfilzomib and dexamethasone (VenKd), resulting in TLS with subsequent renal failure. Repeat marrow biopsy showed no monoclonal plasma cells but extensive fibrosis. Venetoclax was reintroduced after two months with marrow recovery. Venetoclax was titrated from 200 to 400 mg daily alongside IV fluids and allopurinol without TLS recurrence. **Conclusions:** Here, we highlight the importance of risk stratification, dose titration, and TLS prophylaxis with venetoclax use in RRMM.

## 1. Introduction and Clinical Significance

Tumor lysis syndrome (TLS) is a potentially fatal oncologic emergency that can occur when large amounts of tumor cells are lysed and release their contents into the bloodstream [1]. The release of large amounts of potassium, phosphate, and uric acid into the blood induces electrolyte abnormalities which can result in cardiac arrhythmias, seizures, acute renal failure, or multiorgan failure [2]. TLS is most often associated with non-Hodgkin lymphoma, acute myeloid leukemia, and acute lymphoblastic leukemia after the initiation of cytotoxic therapy [3]. Patients with multiple myeloma (MM) are at a low risk of TLS [3]. Most cases of TLS in MM are associated with high-dose chemotherapy and proteasome inhibitors [4,5]. Spontaneous tumor lysis syndrome in multiple myeloma is exceedingly rare, with only a few cases reported in the literature [6,7,8].

MM remains difficult to treat, but new therapies are in development. Venetoclax is one such drug that has been recently studied in the setting of relapsed/refractory multiple myeloma (RRMM). Venetoclax is a potent, orally bioavailable, and selective BCL-2 inhibitor [9] with the potential to induce TLS. However, the protocols for TLS prophylaxis and treatment have been highly variable in clinical trials investigating the drug, as we will briefly review here.

A phase I trial evaluated venetoclax monotherapy for multiple myeloma in 66 patients [10]. TLS prophylaxis involved 1–2 L of oral hydration >72 h before the first dose and at each dose increase. Prophylaxis with uric acid-reducing agents was considered for patients with elevated uric acid levels. Doses were escalated over a two-week lead-in to reach the final doses of 300, 600, 900, or 1200 mg. The maximum tolerated dose (MTD) was not reached, no TLS events were reported, and there were no differences in safety events between doses.

In phase I and II trials of venetoclax plus dexamethasone (VenDex) for t(11;14) RRMM, patients were advised to drink 1–2 L per day before the first dose of venetoclax and to take venetoclax after breakfast [11]. Labs were drawn more frequently for patients at higher risk of TLS (t(11;14) and >50% marrow plasma cell infiltration, or CrCl < 50 mL/min). There was no venetoclax lead-in period for the phase II cohorts of this trial. TLS was observed in 3/51 patients treated with 800 mg daily venetoclax plus dexamethasone. All three cases were grade 3 or 4. TLS was managed with IV fluids (>1 mL/kg/h), medications for hyperkalemia and hyperphosphatemia, and/or dialysis. Similarly, 66 patients were enrolled in a phase I trial of venetoclax plus bortezomib and dexamethasone (VenBd) with venetoclax doses ranging from 100 to 1200 mg [12]. Allopurinol was given >72 h before the first dose of venetoclax for TLS prophylaxis, along with oral hydration. No TLS events occurred in this study, and the MTD of VenBd was not reached.

The phase III BELLINI trial compared 800 mg venetoclax plus bortezomib and dexamethasone (VenBd, n = 193) vs. placebo plus bortezomib and dexamethasone (Bd, n = 96) [13]. TLS prophylaxis was at the investigator’s discretion. Recommendations were for oral hydration >72 h prior to the first dose of venetoclax for all patients and uric acid-reducing agents for patients with high uric acid levels. High-risk patients were monitored more intensely with frequent labs and IV hydration at the investigator’s discretion. TLS was reported in 1/193 patients in the VenBd group and 0/96 patients in the placebo + Bd group. The protocols for TLS management were not described.

Venetoclax plus carfilzomib and dexamethasone for RRMM was investigated in a phase II trial of 43 patients and showed favorable safety and efficacy [14]. TLS prophylaxis was oral and IV hydration >72 h before the first dose. One out of forty-three patients developed TLS on cycle 1 day 2 of 800 mg venetoclax plus 20 mg/m^2^ carfilzomib. The patient who developed TLS in this trial had 60% bone marrow infiltration prior to treatment and t(11;14). The patient was treated with hydration and allopurinol, and treatment resumed four days after the labs normalized.

TLS is a rare occurrence in patients treated with venetoclax mono- or combination therapy, and clear protocols guiding TLS prophylaxis are lacking. Our case report demonstrates the importance of a uniform TLS prophylaxis protocol in treating t(11;14) positive RRMM with venetoclax.

## 2. Case Presentation

We present a 53-year-old gentleman with a past medical history of steroid-induced diabetes mellitus and triple-class refractory multiple myeloma (MM). Prior lines of therapy for MM included radiation therapy; cytoxan, bortezomib and dexamethasone (CyBorD); velcade, revlimid and dexamethasone (VRD); daratumumab, pomalidomide and dexamethasone (dara/pom/dex); and bortezomib, thalidomide, dexamethasone, cyclophosphamide, etoposide and cisplatin (VTD-CEP). A more detailed hematologic history is presented in Supplemental Table S1. Two months prior to presentation the bone marrow biopsy revealed a hypercellular (>90% cellular) marrow with 80–90% plasma cells, reduced trilineage hematopoietic elements, and <5% blasts, indicating disease relapse. Notably, no fibrosis was identified on this biopsy. As per the results of a recent phase II trial [14], the patient was recommended venetoclax plus carfilzomib and dexamethasone (VenKd). For TLS prophylaxis, he was given allopurinol, 300 mg PO daily, was encouraged to orally hydrate himself well for 3 days, and was given 500 mL of IV normal saline 1 h before and after his treatment. He received a single dose of venetoclax 800 mg PO, carfilzomib 20 mg/m^2^ IV, and dexamethasone 40 mg PO five days prior to presentation at our hospital. He was put on levofloxacin, trimethoprim-sulfamethoxazole, and acyclovir for infection prevention.

One day after administering VenKd, the patient presented to his local hospital for fluid overload and acute kidney injury, for which he received one round of hemodialysis (HD), as well as treatment with allopurinol and rasburicase. The kidney failure was attributed to MM, and the patient was discharged to hospice. Five days after the first and thus far only dose of VenKd, the patient presented to our hospital for a second opinion. His chief concerns were shortness of breath, cough, weight gain (37 lbs), and anuria for 24 h before presentation. Vital signs were stable with BP 121/79, T 98.2 °F, HR 83, RR 16, and SpO_2_ 100% on room air. The physical exam was concerning for abdominal distention and bilateral lower extremity edema extending to his thighs. The sudden rise in creatinine, electrolyte profile, and lactate dehydrogenase of >4500 units/dL made acute renal failure (ARF) secondary to TLS the most likely diagnosis, and the patient was hospitalized for aggressive management. ARF was defined as a rapid fall in glomerular filtration rate, manifesting with disrupted salt and water homeostasis and associated increases in serum urea and creatinine. Tumor lysis syndrome was diagnosed using the Cairo-Bishop criteria [15]. Labs on presentation and throughout the admission are summarized in Table 1. The patient was started on diuretics, sevelamer and other supportive care for his ARF along with HD. The patient received five treatments of HD, through day 9 of this 13-day hospitalization. Urine output gradually increased to ~2 L per day. The patient was discharged on 100 mg daily allopurinol and 800 mg sevelamer 3× daily. Serum creatinine and urine output continued to improve post-hospital discharge, heralding gradual restoration of kidney function.

**Figure 1:**
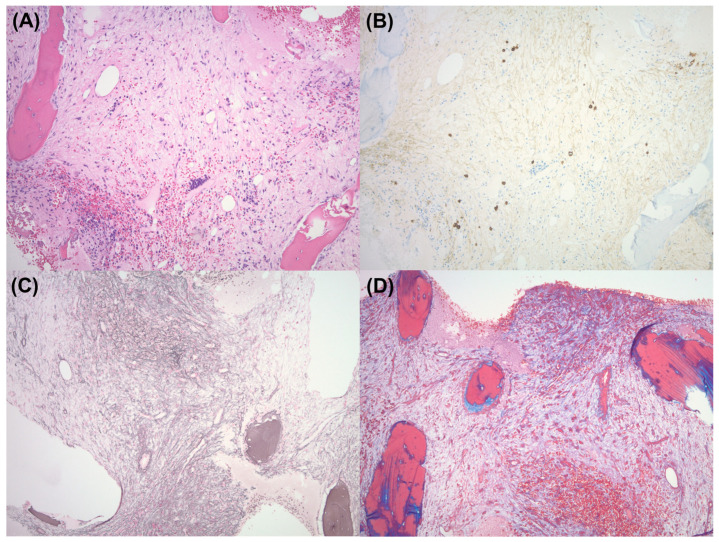
Bone Marrow Biopsy 2 weeks after VenKd therapy: (**A**) Fibrotic, hypocellular marrow with scant islands of hematopoietic elements, predominantly erythroid precursors, and scalloping of bony trabeculae (H&E, 100× magnification). (**B**) Rare residual plasma cells comprising <1% of marrow cellularity, that mark as polytypic by kappa and lambda IHC (CD138 IHC, 100× magnification). (**C**) Diffuse reticulin fibrosis (Reticulin special stain, 100× magnification). (**D**) Focal coarse bundles of collagen fibers consistent with focal WHO grade MF-3 fibrosis in a background of grade MF-2 fibrosis (trichrome stain, 100× magnification).

Our patient was pancytopenic and required four units of packed red blood cells and ten units of platelets during this hospitalization. A repeat bone marrow biopsy taken 2 weeks after treatment while the patient was in the hospital was negative for plasma cell neoplasm. It demonstrated a markedly hypocellular marrow with extensive fibrosis (Figure 1). Flow cytometry showed no significant population of plasma cells.

### Follow-Up and Outcomes

The patient’s health and urine output improved after discharge, with resolution of pancytopenia. Two months later, a repeat bone marrow biopsy showed a normocellular (50% cellular) bone marrow with trilineage hematopoiesis, extensively involved by plasma cell neoplasm at 90%. Flow cytometry revealed a clonal plasma cell population. Reticulin and trichrome stains demonstrated WHO grade 1 fibrosis, suggesting partial resolution of the marrow fibrosis.

The patient restarted venetoclax at 200 mg daily in a controlled hospital setting, aiming for disease control to enable consolidation with high-dose chemotherapy/autologous stem cell transplant or autologous chimeric antigen receptor T-cell therapy. Phosphorus and uric acid levels rose slightly on day three but improved with sevelamer. After 1 week, venetoclax was increased to 400 mg daily with good tolerance. Carfilzomib (20 mg/m^2^ IV/week followed by 27 mg/m^2^ IV twice weekly) and dexamethasone (at 20 mg PO twice weekly) were added outpatient. The patient continued VenKd treatment with good disease response and performance status.

## 3. Discussion

In summary, after one dose of VenKd (800 mg Ven, 20 mg/m^2^ carfilzomib, and 40 mg dex), our patient developed ARF due to TLS, requiring a two-week hospital stay and six rounds of HD. To our knowledge, this is the first case of a MM patient’s bone marrow transitioning from a hypercellular, severely diseased marrow to a hypocellular, fibrotic marrow without monoclonal plasma cells after a single VenKd dose.

TLS is rare in RRMM. Although there are scarcely reported cases of spontaneous TLS in RRMM [6,7], most TLS is associated with myeloma-directed therapy, especially high-dose chemotherapy [4] and proteasome inhibitors (PIs) [5]. Of the commercially available PIs, twice weekly carfilzomib plus dexamethasone was associated with higher rates of TLS in a single-center study from Japan. In this study, 50% of patients treated with carfilzomib plus dex developed TLS with a median onset of 5.5 days after carfilzomib [5]. Venetoclax represents a new group of potent drugs used in RRMM with t(11;14), with the potential to cause TLS. The rising use of these drugs, as monotherapy or combination, necessitates improved TLS risk assessment tools and risk-adapted prophylaxis measures.

TLS associated with venetoclax use is well described in chronic lymphocytic leukemia (CLL). TLS risk assessment tools using absolute lymphocyte count and lymph node size to guide prophylactic measures are implemented in clinical trials utilizing venetoclax [16]. A slow, 5-week dose escalation of venetoclax leads to lower TLS rates compared to high initial doses and rapid escalation [17]. Retrospective data in RRMM show higher disease burden, doses, and drug potency increase TLS rates in RRMM, as do increased LDH, hepatosplenomegaly, and chromosome 13 aberrations [4,5,6]. Studies have also shown worse survival outcomes for patients who develop TLS compared to those who do not, highlighting the disease’s inherently aggressive nature. 

## 4. Conclusions

As such, risk assessment tools that consider disease burden, cytogenetics, and serum LDH should be evaluated and validated with future clinical trials to guide TLS prophylaxis measures, including IV versus oral hydration, the frequency of TLS lab monitoring, the need for slow dose escalation, and the timing and dosing of uric acid lowering therapies.

## Figures and Tables

**Table 1:** Lab values. * Rasburicase administered at outside hospital. A hyphen indicates the lab value was not measured at the corresponding time point.

Day	Date of Infusion	1 Day Post-Infusion	5 Days Post-Infusion	6 Days Post-Infusion (Before HD)	10 Days Post-Infusion (Undergoing HD)	18 Days Post-Infusion	2-Month Follow-Up
Creatinine (mg/dL)	1.0	3.3 (H)	8.71 (H)	10.38 (H)	6.58 (H)	5.82 (H)	1.66 (H)
Potassium (mmol/L)	4.1	7.1 (H)	5.4 (H)	6.2 (H)	3.7	3.4	3.1 (L)
Phosphorus (mg/dL)	-	13.2 (H)	10.7 (H)	12.5 (H)	5.5 (H)	4.1	3.6
Calcium (mg/dL)	9.4	7.4 (L)	6.2 (L)	6.4 (L)	7.4 (L)	7.0 (L)	8.2 (L)
Total Protein (g/dL)	6.2 (L)	7.1	4.8 (L)	-	-	-	6.1
LDH (IU/dL)	-	-	>4500 (H)	4210 (H)	1436 (H)	638 (H)	-
BUN (mg/dL)	14	62 (H)	123 (H)	137 (H)	49 (H)	32 (H)	18
Uric Acid (mg/dL)	-	18.3 (H)	1.2 (L) *	1.8 (L) *	2.7 (L) *	5.2	9.7 (H)

## Data Availability

Data are contained within the article and Supplementary Materials. Further inquiries can be directed to the corresponding author.

## Supplementary Materials

The following supporting information can be downloaded at: https://www.mdpi.com/article/10.3390/reports7040108/s1, Table S1: Timeline of Multiple Myeloma Treatment History.
