# Supplementary material for: Tumor Lysis Syndrome with Venetoclax/Carfilzomib/Dexamethasone for Relapsed/Refractory Multiple Myeloma: A Case Report
Source: Reports (MDPI). 2024 Nov 29;7(4):108. doi: 10.3390/reports7040108 (PMC12199920; doi:10.3390/reports7040108)
Supplement: Supplementary file 1 [file reports-07-00108-s001.zip › reports-3292301-supplementary.pdf]

Supplementary Information:

Supplementary Table S1: Detailed multiple myeloma treatment history, arranged from earliest treatment to most recent treatments leading to the diagnosis of tumor lysis syndrome.

Patient's Age	Time prior to TLS diagnosis	Event:
49	3 years, 4 months	Patient presented with a 2-month history of abdominal pain w/ acute worsening.<br>CT showed diffuse lytic lesions of the spine. Thoracic spine MRI shows 3.6 X 2.1 X 4 cm mass at T8 vertebral body, flattening the cord.
49	3 years, 3 months	Bone marrow biopsy: 12% plasma cells. FISH + for t(11;14), del 13 q. Diagnosis of IgG Kappa MM, standard risk, Duri Salmon Stage IIIA was made. ISS Stage 1.
49	3 years, 3 months	**Radiation Therapy to T8:** 3000 cGy in 10 fractions
49	3 years, 3 months	One cycle of Cytoxan, Bortezomib, Dexamethasone **(CyBorD)**
49-50	3 years, 2 months – 2 years, 10 months	Five cycles of Velcade, Revlimid, Dexamethasone **(VRD)** (4 months)
50-52	2 years, 6 months – 10 months	Revlimid + Dexamethasone maintenance therapy
52	9 months	Patient developed pancytopenia. Repeat bone marrow biopsy: 80% plasma cells. +2, +5, t(11;14), +18, +21. Patient declined ASCT.
52	9 months – 5 months	Three cycles Daratumumab, Pomalidomide, Dexamethasone **(Dara + Pom + Dex**). Patient had transient treatment interruption due to COVID infection.
52	4 months	Repeat bone marrow biopsy: 40% plasma cells
52	3 months – 2 months	Two cycles of Bortezomib, Thalidomide, Dexamethasone, Cyclophosphamide, Etoposide, Cisplatin **(VTD-CEP)**
52	2 months	Patient admitted for neutropenic fever
52	2 months	Bone marrow biopsy showed markedly hypercellular marrow (>90% cellular), composed of 80-90% $CD138^+$, Cyclin $D1^+$ plasma cells, reduced trilineage hematopoietic elements and less than 5% blasts. No bone marrow fibrosis was noted on this biopsy.

53	1 day	A single treatment with **venetoclax, carfilzomib, dexamethasone (VenKd)**<br>Kappa light chains are 192.4mg/L, kappa/lambda ratio 83.65
53	0 days	Hospitalized for TLS requiring HD<br>Patient had worsening pancytopenia requiring blood and platelet transfusions.
